# Aerosol Jet Printed Ion-Selective Electrodes for Potassium Detection

**DOI:** 10.3390/s26103053

**Published:** 2026-05-12

**Authors:** Giorgia Polidori, Emilio Sardini, Mauro Serpelloni

**Affiliations:** Department of Information Engineering, University of Brescia, Via Branze 38, 25123 Brescia, Italy; emilio.sardini@unibs.it (E.S.); mauro.serpelloni@unibs.it (M.S.)

**Keywords:** aerosol jet printing, ion-selective electrodes, potassium detection, solid-contact sensors, MWCNT transducer, PVC ion-selective membrane, single-interferent response tests, printed electronics

## Abstract

This work evaluates a potassium ion-selective electrode (K-ISE) fabricated using Aerosol Jet Printing (AJP) and compares its performance with that of a commercial K^+^-selective electrode (KION). Both sensors exhibit near-Nernstian behavior, with average sensitivities of 57.91 ± 5.07 mV/dec for the AJP device and 57.28 ± 5.07 mV/dec for the commercial electrode, confirming a near-Nernstian K^+^ response over the tested concentration range. Single-interferent response tests demonstrate that AJP-printed electrodes provide a more stable and less sensitive response to sodium interference (24.37 ± 1.39 mV/dec) compared to KION (33.95 ± 8.95 mV/dec), while showing comparable NH_4_^+^ response and a slightly higher response toward urea than KION. Morphological analysis (OM and SEM) reveals that AJP enables smoother, more homogeneous films and improved control over the transducer/membrane interface. Unlike previous studies, this work presents a direct experimental comparison between AJP-fabricated and commercial ISEs under controlled interference conditions relevant to agricultural and environmental matrices. Although the AJP sensors exhibited near-Nernstian behavior and fast response times, their reproducibility was lower than that of the commercial electrodes (RSD = 30.12% vs. 18.45%), indicating that further optimization of the printing and membrane deposition processes is required.

## 1. Introduction

The rapid growth of additive manufacturing and printed electronics has enabled the development of miniaturized and low-cost electrochemical sensors. This technological evolution is driven by the increasing need for portable and real-time analytical tools capable of operating outside traditional laboratory environments, supporting applications in environmental monitoring, precision agriculture, wearable health systems, and industrial process control [1,2].

Within this context, ion-selective electrodes (ISEs) have emerged as a powerful class of sensors for real-time monitoring of ionic species in environmental, biomedical, and agricultural applications [3]. Potassium detection is particularly relevant because K^+^ plays a central role in plant physiology, nutrient uptake, osmotic regulation, and crop productivity, as well as serving as an indicator of water quality and physiological homeostasis in environmental and biomedical systems [4].

Traditional analytical methods—based on sample extraction and spectroscopic or chromatographic analysis—provide accurate results but require expensive instrumentation, trained personnel, and are unsuitable for in situ or continuous measurements [5,6].

This limitation has accelerated interest in solid-contact ion-selective electrodes (SC-ISEs), which combine portability, high sensitivity, and low-power operation [7]. SC-ISEs introduce a conductive transducer between the ion-selective membrane and the electronic contact, improving charge transfer and potential stability [8]. Nanostructured materials such as carbon nanotubes, graphene, and conductive polymers (e.g., PEDOT:PSS) have further improved charge transfer and potential stability, enabling robust and miniaturized devices suitable for long-term monitoring [9,10,11,12].

Recent advances in printed electronics have further accelerated the development of customizable and low-cost ISEs.

Screen printing remains the most widely adopted technique due to its high batch reproducibility and mechanical robustness [13,14]. However, its limited resolution and control over thin-film microstructure constrain sensor performance, particularly at the transducer/membrane interface, where morphology strongly influences potentiometric stability and drift [15,16,17].

In contrast, Aerosol Jet Printing (AJP) has emerged as a promising alternative, offering high spatial resolution (10–50 µm), contactless deposition, and compatibility with nanomaterial-based inks [18,19,20]. AJP enables precise control over layer thickness, lateral geometry, and material distribution, allowing the engineering of finely structured interfaces that enhance ion-to-electron transduction [21].

Its ability to deposit uniform films on both planar and non-planar substrates makes AJP particularly suitable for flexible and wearable ion-sensing platforms.

Several studies have demonstrated that additive manufacturing techniques can improve film uniformity and interfacial contact, leading to enhanced potentiometric stability and reduced drift [22,23,24,25]. Despite these advances, the influence of fabrication techniques on the morphological and electrochemical behavior of K^+^-selective electrodes remains insufficiently explored, especially under realistic conditions involving ionic interference, variable matrices, and low ionic strength [26,27,28].

In particular, the response of AJP-printed K^+^ electrodes in the presence of common interferents such as Na^+^, NH_4_^+^, and urea has not been systematically evaluated, despite their relevance in agricultural and environmental matrices [29].

This work addresses this gap by providing a direct experimental comparison between AJP-fabricated and commercial ion-selective electrodes under single-interferent conditions relevant to agricultural and environmental matrices. Through morphological (OM and SEM) and electrochemical analyses, the study investigates whether the advantages of AJP in terms of deposition control and film uniformity translate into measurable improvements in sensitivity, stability, repeatability, and single-interferent response tests.

By examining both metrological performance and interference robustness, this work contributes to establishing AJP as a viable manufacturing route for next-generation wearable, flexible, and field-deployable ion-sensing platforms.

## 2. Materials and Methods

### 2.1. Sensor Description and Fabrication

**Figure 1:**
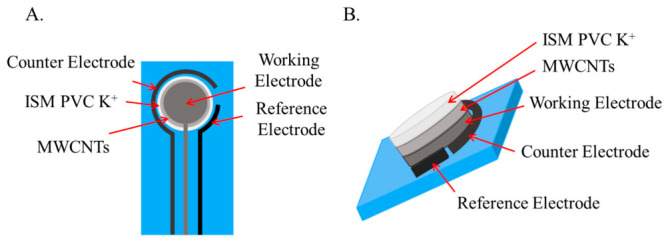
Detailed explanation of the elements of (**A**). frontal sensors 2D image and (**B**). lateral sensors 3D image.

Two types of potassium ion-selective electrodes (ISEs) were studied to compare their electrochemical performance. In general, electrochemical sensors consist of various electrodes, shown in Figure 1, to which an interface material (Multi-Wall Carbon Nanotubes—MWCNTs) and an ion-selective membrane specific for potassium detection (ISM—PVC K^+^) have been printed. The PVC-based potassium-selective membrane was prepared using a standard valinomycin formulation obtained by dissolving 5.142 × 10^−3^ M potassium ionophore, 4.174 × 10^−3^ M NaTPB, 433.25 × 10^−3^ M DOS and PVC (9.371 wt%/vol%) in cyclohexanone. The resulting K^+^ mixture was vigorously stirred for one hour to create a uniform membrane solution, following the composition reported in [30].

The sensors used in this study were commercial electrodes and electrodes modified in the laboratory by Aerosol Jet Printing (AJP). The investigated sensor types are summarized in Table 1.

The first type of sensors used consisted of commercial electrodes DRP-110KION (Metrohm DropSens, Oviedo, Spain), screen-printed carbon-based electrodes, supplied with a PVC ion-selective membrane for K^+^. Screen printing is a mature and widely used technique for disposable electrochemical sensors because it offers high batch reproducibility, as each device is produced with the same mesh, ink, and polymerization conditions, good mechanical robustness, and high pattern fidelity. On the other hand, this type of printing has limited control over nanoscale features and surface microstructure, as the morphology is determined by the carbon ink formulation and mesh size. The ion-selective membrane is typically cast or printed in a single step, with limited thickness control [15]. The manufacturer does not disclose the exact composition of the membrane; the presence of conductive additives (e.g., PEDOT, CNTs) is assumed but not guaranteed, so there is limited ability to customize or optimize the transducer/membrane interface. These characteristics ensure stable basic performance but limit the possibility of adjusting the sensitive layers.

The second type of electrodes consisted of devices fabricated using Aerosol Jet Printing (AJP) with an Optomec AJ-300 system (Optomec Inc., Albuquerque, NM, USA). This additive manufacturing technique was used to deposit both the MWCNT-based solid contact layer and the PVC-based potassium-selective membrane onto the same DRP-C110 (Metrohm DropSens, Oviedo, Spain) substrates. Using the AJP pneumatic configuration, a uniform layer of MWCNT ink was first printed under optimized conditions, producing a continuous conductive network with minimal agglomeration. After the drying process of the MWCNTs layer, the ion-selective PVC membrane was then deposited using the same printing mode, with parameters adjusted to account for the higher viscosity of the membrane formulation. This sequential deposition enabled the formation of homogeneous films with precise control over membrane thickness and good reproducibility.

In pneumatic AJP, the ink is atomized into a fine aerosol inside the atomizer and transported to the nozzle by a primary gas flow. A coaxial coating gas then concentrates the aerosol flow into a highly collimated jet that reaches the substrate completely contact-free.

This mechanism supports stable aerosol formation even for inks containing nanomaterials and enables the deposition of functional layers with micrometric resolution. The process provides excellent control of thickness, edge definition, and lateral dimensions, making it suitable for creating uniform sensor architectures on flat or slightly three-dimensional surfaces [31].

The optimized parameters for printing the MWCNT transducer included sheath flow rate 600 sccm, atomizer gas flow 850 sccm, exhaust gas flow 650 sccm, 750 µm nozzle diameter, plate temperature 60 °C, and a printing speed of 3 mm/s. These conditions produced a uniform conductive network with minimal agglomeration and uniform film coverage.

The following printing speeds and flow rates were used for the PVC-based membrane: sheath flow rate 1800 sccm, atomizer gas flow 1500 sccm exhaust gas flow 1300 sccm, 750 µm nozzle, plate temperature 20 °C, and a printing speed of 3 mm/s, in order to obtain a smooth and uniform ion-selective film with minimal edge thickening.

**Figure 2:**
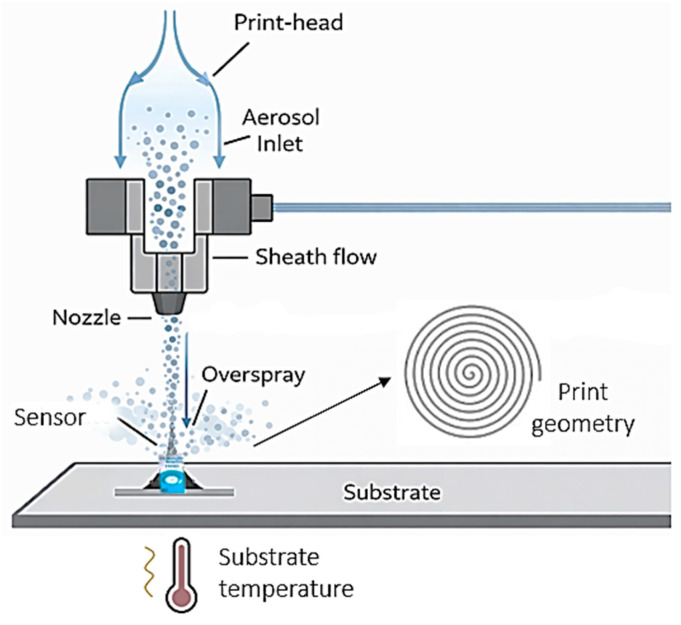
Represents method and deposition by Aerosol Jet Printing (AJP) of the sensitive layers using a circular/spiral printing geometry.

To ensure uniform coverage of the active electrode area, a circular spiral deposition pattern was chosen for both the transducer and the membrane layers (Figure 2). This geometry improves film uniformity and overall sensor reproducibility.

A total of 30 sensors were used in this study: 15 commercial KION electrodes (from the same production batch) and 15 AJP-printed electrodes fabricated under identical printing conditions.

### 2.2. Morphological Characterization

The aim of the morphological analysis was to evaluate the surface morphology of the deposited films. The surface morphology of the ion-selective electrodes was analyzed using optical microscopy (OM) and scanning electron microscopy (SEM) in order to evaluate the quality and homogeneity of the conductive and ion-selective layers obtained with different manufacturing techniques (commercial screen printing and aerosol jet printing).

Optical microscopy images were acquired using an NB50T microscope (Orma Scientific, Sesto San Giovanni, Milan, Italy) equipped with a trinocular continuous-zoom system (0.8×–5×). Images were captured at low magnification to visualize the entire electrode area, with the optical zoom set to 0.8× and an additional 2× digital zoom factor, providing a field of view sufficient to visualize the entire electrode area. OM analysis was mainly used to inspect surface uniformity, the presence of macroscopic defects, and the overall quality of the deposition of conductive and ion-selective layers produced using the two different fabrication methods.

Although optical microscopy does not provide the nanoscale resolution necessary to analyze the internal morphology of the membrane or the distribution of carbon nanostructures, the microstructure of the sensors was subsequently analyzed using a high-resolution scanning electron microscope.

SEM images were obtained using a PHENOM XL G2 scanning electron microscope (Thermo Fisher Scientific, Waltham, MA, USA) operated at 5 kV, with scale bars of 80 µm and 300 µm respectively. SEM analysis revealed clear morphological differences between the two types of sensors, although it is not possible to evaluate the distribution of the ionophores and the membrane.

### 2.3. Measurement Setup

Electrochemical measurements were performed using a PalmSens4 potentiostat (PalmSens BV, Houten, The Netherlands) in a three-electrode configuration (working electrode = printed/commercial sensor, reference = integrated Ag/AgCl, counter electrode = carbon trace).

Prior to use, all sensors were preconditioned for 15 min in a 10 mM KCl solution to stabilize the ion-selective membrane and reduce initial signal drift. Preconditioning is a standard requirement for both commercial and laboratory-made ion-selective electrodes, as it promotes membrane equilibrium, improves the phase boundary potential, and minimizes instability in the initial phase. Similar procedures are reported in the literature; for example, Novell et al. preconditioned their solid-contact ISEs for 20 min in a 10^−4^ M primary ion solution [32]. Although the specific conditions may vary depending on the composition of the membrane, plasticizer, and solid-contact material, the basic logic remains the same [3].

In this work, the selected preconditioning parameters were optimized experimentally: several preliminary tests showed that 15 min in 10 mM KCl provided the most stable and reproducible potential for the materials and membrane formulation used.

**Figure 3:**
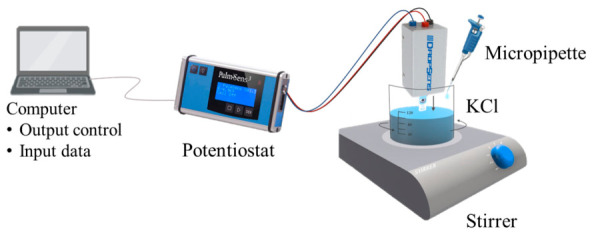
Electrochemical measurements setup.

The measurement protocol was carried out using a 100 mL beaker (Figure 3), used both for sensor testing and interference tests. All potentiometric measurements were performed at room temperature using a PalmSens4 potentiostat (PalmSens BV, Houten, The Netherlands) in a three-electrode configuration:

- working electrode: printed or commercial K^+^ selective sensor;
- reference electrode: integrated Ag/AgCl;
- counter electrode: carbon trace.

Electrochemical tests were conducted in standard KCl solutions ranging from 10^−6^ M to 10^−2^ M, using incremental additions of KCl. The potential difference (E) was recorded after signal stabilization (typically 100 s) and plotted as a function of log[K^+^].

Single-interferent response tests were performed by evaluating each interferent (Na^+^, NH_4_^+^, and urea) individually, in the absence of potassium ions. This single-interferent protocol allowed quantifying the isolated effect of each species on the electrode potential without contributions from K^+^. All experiments were conducted under constant stirring and at room temperature. Figure 3 illustrates the electrochemical measurement setup.

### 2.4. Preparation of Standard Solutions for Tests

The sensors were tested in distilled water using standard KCl solutions at concentrations ranging from 10^−6^ M to 10^−2^ M. The selected concentration range reflects the typical operating range of solid contact ion-selective electrodes (SC-ISEs), between 10^−6^–10^−1^ M before reaching the upper detection limit due to membrane saturation. Concentrations below 10^−6^ M often fall below the lower detection limit due to the increased influence of noise and water autoionization, while values above 10^−2^–10^−1^ M saturate the PVC-based membrane. Therefore, the chosen range ensures that the sensor operates in a linear, reliable, and physically meaningful manner; in particular, in agricultural and environmental systems [33]. Indeed, potassium is generally present in soil solution at concentrations ranging from 0.1 to 5 mM (10^−4^–5 × 10^−3^ M), depending on soil composition, fertilization practices, and plant uptake dynamics.

The stock solution (0.1 M) was prepared by dissolving KCl in distilled water and weighing the salt using an analytical balance; from this, the diluted working solutions were obtained by serial dilutions (according to the relationship C_1_V_1_ = C_2_V_2_) using standard microvolume micropipettes.

The standard solutions were added sequentially to a 100 mL beaker of distilled water (Table 2) and the potential was recorded upon reaching stability (average time: ~100 s). The response of the sensors was then analyzed by plotting the measured potential against the logarithm of the K^+^ concentration to construct the curve.

The accuracy of the final concentrations was ensured by the use of high-precision procedures throughout the preparation of the standard solutions. The KCl stock solution was weighed using an analytical balance with a resolution of ±0.1 mg, and all dilutions were performed using ISO-certified micropipettes. The serial addition protocol in the beaker further reduces volumetric error, as each incremental change in concentration depends on small, highly controlled pipetted volumes.

### 2.5. Interference Test

Interference experiments were conducted by progressively increasing the concentration of the interfering species in the absence of potassium ions [3]. This ensured that the individual effect of each interferent on the electrode potential could be evaluated without the contribution of K^+^.

A 100 mL aliquot of distilled water (K^+^ = 0 M) was used as the initial matrix. The interferents chosen were those typically found in soil, which are also used as fertilizers. The selected interferents—NaCl, NH_4_OAc, and urea—are representative of common soil and fertilizer components: sodium is known to interfere with K^+^-selective membranes, ammonium can compete with potassium for ionophore binding sites, and urea may alter the physicochemical properties of the aqueous matrix.

These interferents were gradually added until final concentrations between 10^−6^ M and 10^−2^ M were reached, following the same incremental-addition protocol used for potassium. The potential was recorded after stabilization (~100 s).

For each type of sensor (commercial and AJP), the deviation of the measured potential from the baseline signal in pure distilled water was plotted as a function of the logarithm of the interferent concentration. This procedure allowed quantification of the impact of each interferent, comparison of the robustness of the two fabrication approaches, and simulation of single-interferent conditions relevant to agricultural and environmental matrices, without the confounding effect of potassium. These experiments were intended as controlled single-interferent response tests rather than as a formal determination of potentiometric selectivity coefficients.

## 3. Experimental Results

### 3.1. Optical Characterization

**Figure 4:**
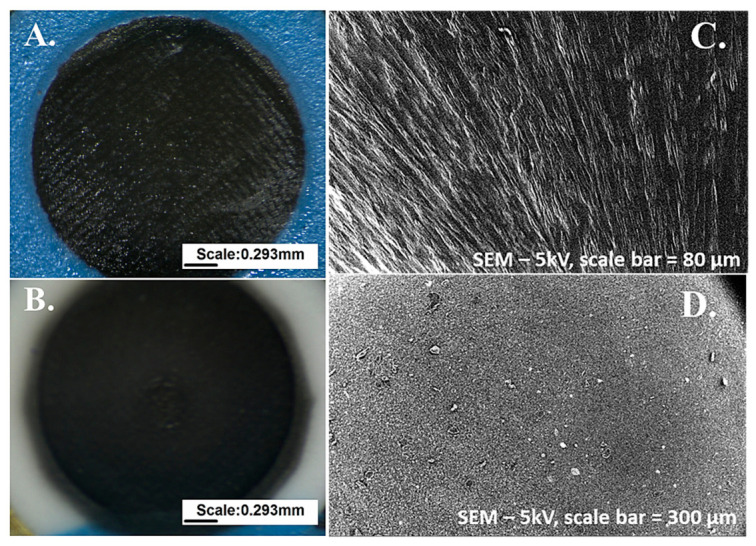
Optical microscopy (OM) and scanning electron microscopy (SEM) images of the commercial KION electrode and the AJP-printed electrode. (**A**) OM image of the commercial screen-printed electrode showing the carbon microgranular structure and the ion-selective membrane. (**B**) OM image of the AJP-printed electrode showing a smoother and more homogeneous membrane surface. (**A**,**B**) Images were captured at low magnification, with the optical zoom set to 0.8× and an additional 2× digital zoom factor, providing a field of view sufficient to visualize the entire electrode area. (**C**) SEM image of the commercial electrode (scale bar: 80 µm) highlighting the compact microgranular carbon layer and the non-uniform membrane coverage. (**D**) SEM image of the AJP-printed electrode (scale bar: 300 µm) showing a continuous, finely structured membrane with well-distributed MWCNT networks.

Optical microscopy (OM) analysis revealed that:

In commercial screen-printed electrodes (Figure 4A), OM allowed clear visualization of the underlying carbon microgranular structure and the relatively uniform dispersion of the ion-selective coating applied during industrial manufacturing.

In sensors printed with AJP (Figure 4B), observations under an optical microscope showed visibly more homogeneous films, with smoother macroscopic surfaces and fewer deposition defects.

SEM analysis further highlighted significant morphological differences: commercial KION electrodes, as shown in Figure 4C, had a compact surface with a microgranular structure typical of screen-printed carbon layers, covered with a relatively smooth, though not fully uniform, ion-selective coating.

In contrast, as shown in Figure 4D, the AJP-printed sensors exhibited a homogeneous, continuous, and finely structured film with well-distributed MWCNT networks embedded in the polymer matrix. Controlled aerosol deposition resulted in smoother topography and reduced membrane thickness variability.

These observations are consistent with reports in the literature on printed solid-contact ion-selective electrodes, where the greater film uniformity and interfacial contact achieved through additive manufacturing are correlated with greater potentiometric stability and lower signal drift [3,11].

### 3.2. Tests of Sensors in Distilled H_2_O

The sensors (commercial KION and AJP) were analyzed according to the addition scheme shown in Table 2 (Section 2).

**Figure 5:**
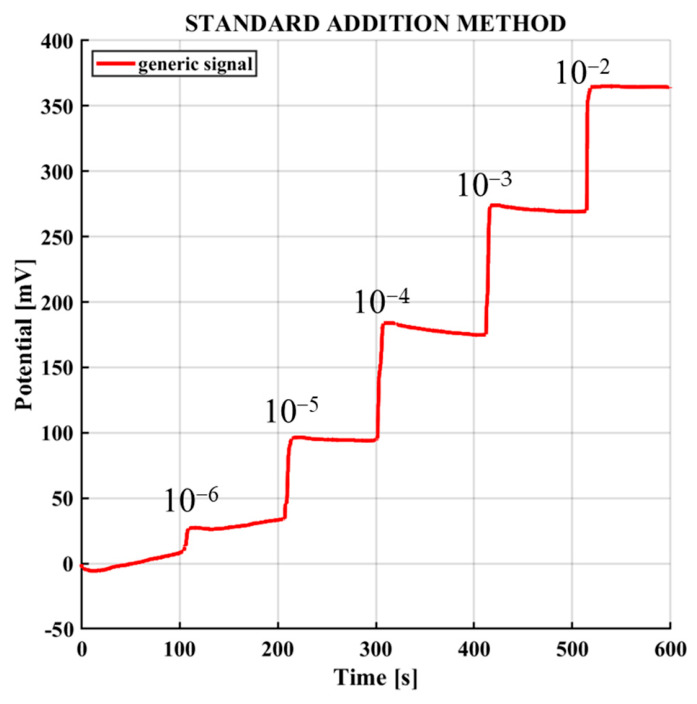
Signal relating to the standard potassium addition method with AJP sensors, in a voltage vs. time graph.

Figure 5 shows a representative voltage-time response obtained during the test procedure using the standard addition method [34,35]. All devices exhibited an approximately linear trend in the range 10^−6^–10^−2^ M, based on the steady-state potentials extracted from the time-dependent response, which is consistent with the well-established linear range of valinomycin-based potassium ion-selective electrodes reported in the literature [36,37,38]. In this example, corresponding to an AJP sensor, the potential remains stable for approximately 100 s after each addition, followed by a gradual change associated with the increase in K^+^ concentration. The same experimental protocol and response behavior were observed for all tested sensors, including commercial and AJP-printed electrodes.

Repeatability was initially evaluated by performing consecutive measurement cycles on the same sensor. However, commercial screen-printed electrodes are designed as disposable devices and are not intended for multiple reuses. During preliminary experiments, a progressive decrease in sensor response was observed after several measurement cycles, most likely due to membrane degradation induced by washing and reuse procedures.

**Figure 6:**
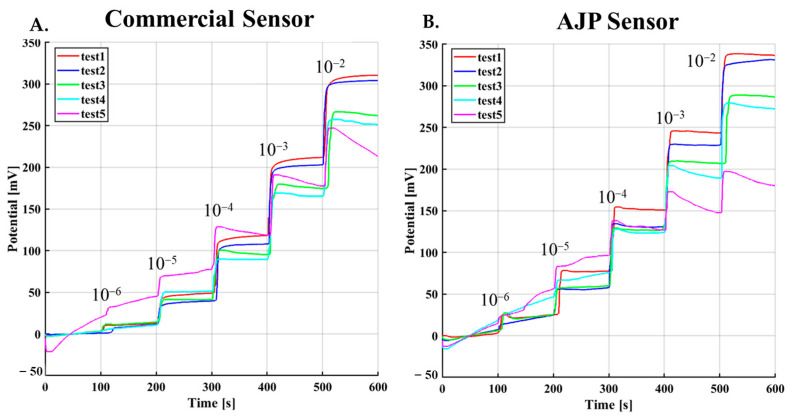
Repeatability analysis of commercial KION and AJP-printed electrodes over five consecutive measurement cycles. (**A**) Potential response of the commercial KION sensor showing signal decay after the fourth cycle due to membrane degradation. (**B**) Potential response of the AJP-printed sensor showing stable behavior over four cycles and degradation at the fifth measurement.

As illustrated in Figure 6, a systematic signal decay was observed starting from the fifth measurement cycle. For this reason, only the first four cycles were considered for the repeatability analysis, while the fifth cycle was excluded as it reflects sensor degradation rather than intrinsic measurement variability. This behavior is also associated with the repeated washing and drying phases applied between measurements, which progressively compromise the integrity of the ion-selective membrane, leading to its deterioration and partial saturation of the ionophore, as shown by test 5 in Figure 6. It is also worth noting that these sensors are inherently designed for single-use applications [39], which further supports the performance evaluation to a single triplicate measurement.

Table 3 summarizes the metrological performance of the sensors over four consecutive calibration cycles. The fifth run was excluded from the statistical analysis as it exhibited a significant loss in sensitivity, likely due to membrane saturation or the gradual leaching of the ionophore into the aqueous solution: a common phenomenon in solid-contact ion-selective electrodes (SC-ISEs). The sensitivity of all sensors was evaluated in terms of 59.16 mV/dec (Nernstian slope) for monovalent ions at room temperature (25 °C), according to IUPAC recommendations [40]. The obtained average slope of 57.28 ± 5.07 mV/dec for the commercial sensor and 57.91 ± 5.07 for the AJP sensor demonstrates a near-Nernstian behavior, consistent with other solid-contact ISEs reported in the literature [37]. The AJP-printed sensors demonstrated slightly improved sensitivity compared to the commercial sensors, indicating that the Aerosol Jet Printing technique provides a high-quality interface between the transducer and the selective membrane. The standard deviation of the various slopes calculated over four cycles (SD) was used as the repeatability parameter. The SD values, shown in Table 3, reflect the variability in the response of the various sensors and also summarize the sensitivity values obtained for the different sensor manufacturing approaches. The SD values reported in Table 3 describe the variability between consecutive measurements and enable comparison among the different fabrication approaches.

The performance evaluation reported in Table 3 provides a comparative overview of the analytical behavior of the commercial and AJP-based electrodes. While the AJP sensors exhibit acceptable sensitivity and linearity, these metrics alone are not sufficient to fully establish equivalence with the commercial electrodes. Therefore, the results should be interpreted as indicative rather than conclusive, and additional performance parameters are considered in the following sections.

The Relative Standard Deviation (RSD), defined as the standard deviation divided by the mean and expressed as a percentage, showed comparable values for both types (8.86% for KION and 8.75% for AJP). These data indicate comparable short-term within-sensor repeatability under the adopted repeated-cycle protocol; however, device-to-device reproducibility remains lower for AJP sensors, as shown in Table 4.

Moreover, AJP sensors showed a faster average t_90_ (23.53 s) compared to KION sensors (26.44 s), suggesting more efficient ion diffusion at the electrode interface.

**Figure 7:**
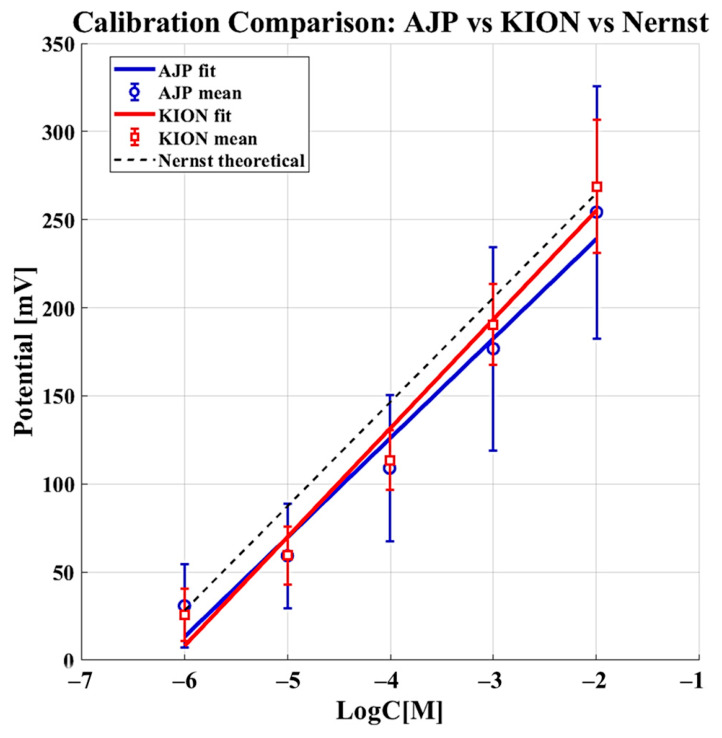
Reproducibility curves for KION (red) and AJP (blue) single-use sensors. The calibration curves shown were obtained from 15 independent sensors for each manufacturing method (*N* = 15 for KION and *N* = 15 for AJP), each measured only once to simulate single-use conditions. The experimental responses are compared with the theoretical Nernst calibration line (black).

To evaluate the potential of these devices as single-use sensors, their reproducibility was assessed by testing a total of 30 sensors (*N* = 15 for KION and *N* = 15 for AJP), with a single measurement taken for each sensor to reflect their intended single-use application. The resulting reproducibility curves are shown in Figure 7, which displays the calibration curves obtained from the 15 sensors for each manufacturing method (KION and AJP) and compares them with the theoretical Nernst response.

The RSD values obtained for the AJP-based electrodes are higher than those of the commercial sensors, indicating reduced short-term stability and greater signal variability. This suggests that, although the AJP electrodes are functional and responsive, their reproducibility is still inferior to that of the commercial counterparts. These results highlight the need for further optimization of the printing process and membrane deposition to improve stability.

A number of physicochemical factors commonly reported for solid-contact ion-selective electrodes (SC-ISEs) may also contribute to the increased variability observed in AJP-printed devices. Fluctuations in the solid-contact capacitance and interfacial charge-transfer processes [41], local inhomogeneities in the conductive layer affecting the pseudocapacitive behavior [42], and micro-scale variations in membrane thickness or morphology [43] are known to influence short-term stability and repeatability. In the case of AJP sensors, additional variability may arise from small differences in printed layer thickness, surface roughness, or ink deposition uniformity, which are intrinsic to the current stage of process optimization. These considerations help contextualize the higher RSD values and indicate potential directions for improving the reproducibility of AJP-based SC-ISEs.

The Standard Deviation and RSD values in Table 4 are notably higher than those in Table 3. The AJP sensors showed a reproducibility RSD of 30.12% and the observed RSD is related to the optimization phase of the automated deposition of the selective membrane. Unlike the mature industrial screen-printing used for KION sensors, the AJP parameters for the polymer membrane (such as atomization flow and ink viscosity) are still being refined. Despite this variability, the AJP electrodes still exhibit favorable kinetic behavior (lower t90) and maintain a consistent ND%, indicating that the electrochemical interface remains efficient even though reproducibility requires further optimization.

The Nernst Deviation (ND%, accuracy) remained low for both categories (<5%), confirming that the electrochemical response remains fundamentally consistent with Nernstian theory across different batches.

The AJP sensors maintained a lower average response time (21.10 s) than the commercial sensors (31.14 s) during the reproducibility tests. This suggests that the AJP approach consistently produces electrodes with faster kinetic responses, which is a significant advantage for rapid diagnostic applications.

### 3.3. Effects of Interferents

**Figure 8:**
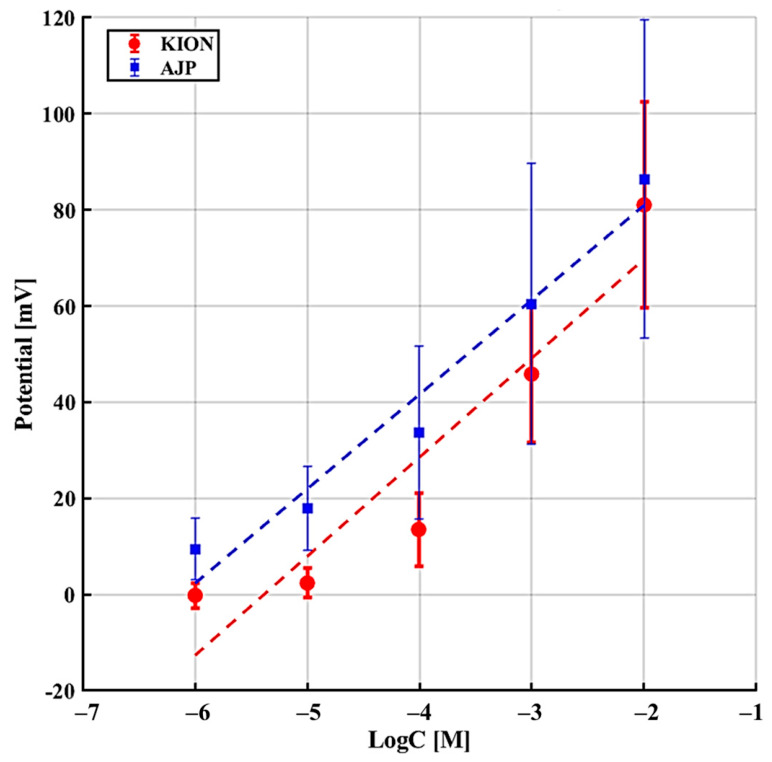
Sensor response in the presence of NH_4_^+^ as interferent.

**Figure 9:**
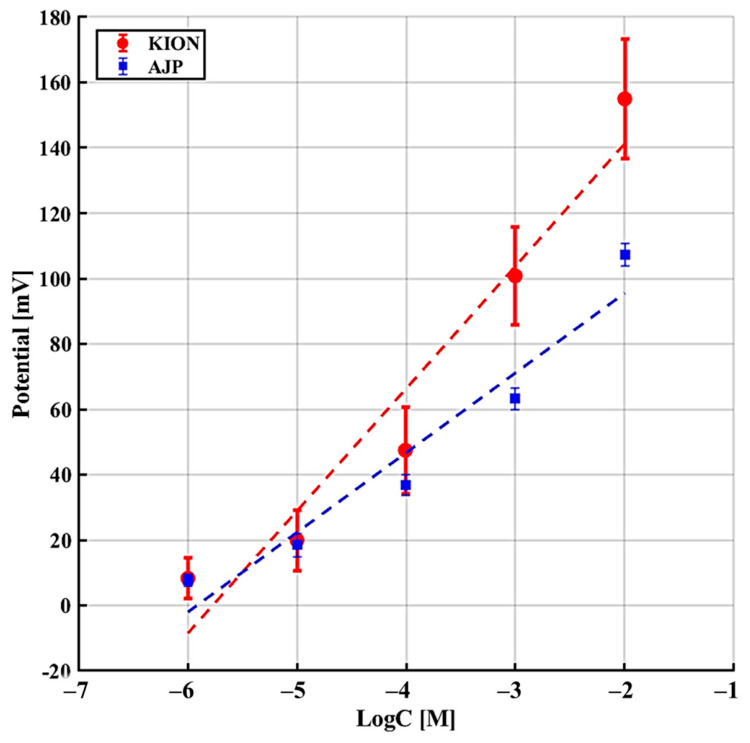
Sensor response in the presence of Na^+^ as interferent.

**Figure 10:**
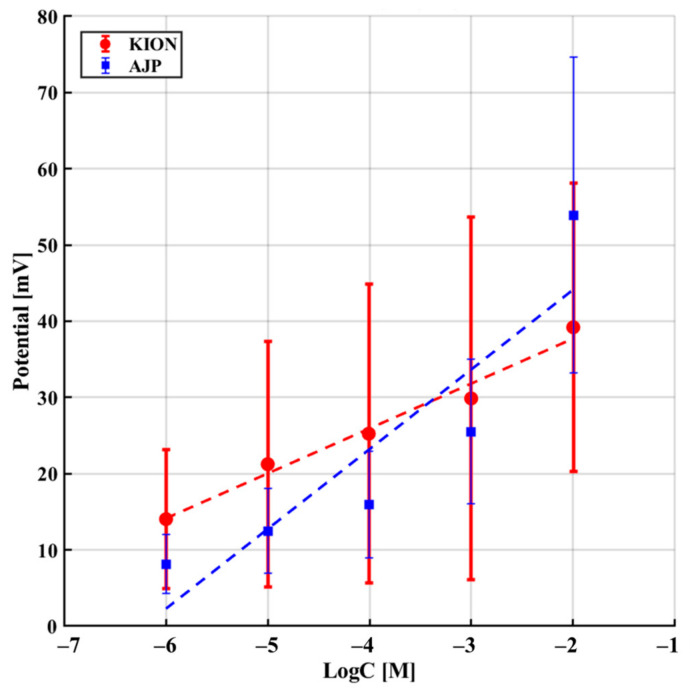
Sensor response in the presence of urea as interferent.

The interference experiments involving Na^+^, NH_4_^+^, and urea were conducted according to the sequence described in Table 2 (Section 2). Figure 8, Figure 9 and Figure 10 illustrate the potential response as a function of interferent concentration, where each data point represents the average of multiple trials with the corresponding standard deviation. As shown in Table 5, both sensors demonstrate a significantly lower sensitivity to interferents compared to the ideal Nernstian slope for potassium (≈59 mV/dec), confirming their single-interferent response tests. Specifically, the AJP sensor exhibited a more stable and less sensitive response to sodium (24.37 ± 1.39 mV/dec) compared to the KION sensor (33.95 ± 8.95 mV/dec). This result is particularly significant because Na^+^ and K^+^ are both monovalent ions with similar chemical behaviors, suggesting that AJP technology offers improved resistance to sodium interference. Regarding urea, the AJP sensor showed a slightly higher sensitivity (10.46 mV/dec) than KION (5.89 mV/dec), but maintained a comparable standard deviation, indicating good reproducibility.

**Figure 11:**
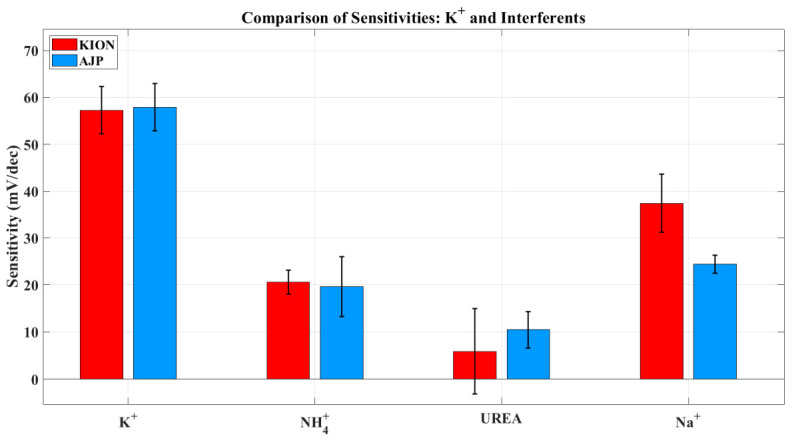
Bar plot comparing the sensitivities of KION and AJP electrodes toward K^+^ and the main interfering species (NH_4_^+^, urea, and Na^+^). The plot highlights the expected selectivity pattern of valinomycin-based membranes (K^+^ >> Na^+^ > NH_4_^+^ >> urea) and shows the reduced Na^+^ sensitivity of AJP-printed electrodes compared to commercial KION sensors.

To better visualize the single-interferent response trends, the sensitivities obtained from the linear regression of the interference curves were summarized in a grouped bar plot (Figure 11). This representation allows a direct comparison between the two fabrication approaches for each analyte. Both sensors show the highest undesired response toward Na^+^, consistent with the known cross-affinity of valinomycin-based membranes. Notably, AJP-printed electrodes exhibit a lower Na^+^ sensitivity than commercial KION sensors, indicating improved resistance to sodium interference despite their higher overall variability.

The results reported in Table 5 further support this interpretation. While the AJP-based electrodes exhibit a measurable and consistent potentiometric response, their behavior under repeated measurements and interference conditions reveals performance variations that are more pronounced than those observed for the commercial sensors. These deviations indicate that, although the AJP approach is promising, its robustness and reproducibility are not yet fully comparable to those of industrially manufactured electrodes. Overall, Table 5 highlights both the functional potential of the AJP sensors and the need for further optimization of the printing and membrane-deposition processes to achieve performance uniformity across batches.

Moreover, the higher sensitivity of the AJP sensor toward urea can be attributed to physicochemical factors commonly reported for solid-contact ion-selective electrodes (SC-ISEs). Neutral or weakly interacting species such as urea may influence the phase-boundary potential depending on membrane morphology, polymer thickness, and the characteristics of the solid-contact/membrane interface [41]. AJP-printed membranes may present slightly higher porosity or micro-scale heterogeneity compared to the commercial KION electrodes, which could facilitate a greater degree of urea partitioning or interaction at the membrane surface [43]. These effects are consistent with the approximately two-fold difference in urea sensitivity observed between the two sensor types and highlight how fabrication-related microstructural differences can influence interferent responses.

The adoption of Aerosol Jet Printing (AJP) for the fabrication of ion-selective electrodes represents a significant shift from traditional subtractive or manual deposition techniques. The dynamic performance advantages observed in the AJP-printed sensors, specifically the reduced response time and enhanced potential stability, is deeply rooted in the unique capabilities of this additive manufacturing process.

Unlike traditional commercial manufacturing, which often relies on standardized, bulkier architectures, AJP allows for the high-resolution deposition of both conductive inks and sensing membranes on a micrometer scale. This spatial precision enables the development of miniaturized sensing arrays without the loss of metrological quality, facilitating the integration of K-ISEs into multi-analyte “Lab-on-a-Chip” platforms and wearable devices [44,45].

The AJP nozzle atomizes the membrane solution into a fine mist of droplets, which are then focused by a sheath gas. This process ensures a highly controlled and uniform film thickness. A thinner and more homogeneous membrane, as evidenced by the t90 results, significantly reduces ion diffusion resistance and eliminates structural defects, which is common in drop-casting or inkjet printing [46].

As an additive process, AJP deposits material only where required, significantly reducing the waste of expensive chemical reagents and ionophores. Furthermore, the non-contact nature of AJP allows for printing on a wide variety of substrates, including flexible polymers and non-planar surfaces, opening new possibilities for conformal sensors that can adapt to the complex geometries of the human body [45,47].

## 4. Discussion

The experimental results confirm that AJP-printed potassium sensors represent a promising fabrication route, although their batch-to-batch reproducibility remains lower than that of commercial electrodes. A central objective of this study was to assess whether the additive manufacturing process could compromise the electrochemical performance of the ion-selective membrane. The results clearly demonstrate that AJP deposition preserves the functional properties of the membrane while providing improved control over film morphology and interfacial quality.

A key advantage of the AJP-fabricated electrodes emerged in the response-time analysis (Table 3). AJP sensors exhibited lower average t_90_ values than commercial KION electrodes. In ion-selective sensing, response time is governed by ion diffusion through the membrane and by the establishment of the electrical double layer at the transducer interface. The high-resolution, contactless nature of AJP enables the deposition of thinner and more homogeneous membranes, effectively reducing the diffusion path length for K^+^ ions and facilitating faster equilibration. In addition, the uniform distribution of MWCNTs within the solid-contact layer likely enhances charge transfer kinetics and reduces interfacial capacitance dispersion, further contributing to the faster dynamic response. This behavior is particularly advantageous for real-time monitoring and lab-on-chip applications, where rapid signal stabilization is essential.

Single-interferent response tests (Table 5) further highlight the benefits of AJP processing. Both AJP and KION sensors displayed sub-Nernstian slopes in the presence of interfering species, as expected for well-performing K^+^-selective electrodes. However, the AJP sensors showed a markedly more stable and less sensitive response to sodium (24.37 ± 1.39 mV/dec) compared to the commercial electrodes (33.95 ± 8.95 mV/dec). This reduced variability suggests the presence of a more homogeneous membrane structure and a more controlled ion-exchange interface. In conventional screen-printed sensors, local variations in membrane thickness and incomplete adhesion can lead to heterogeneous ion flux and potential instabilities. In contrast, the layer-by-layer and highly controlled deposition of AJP likely minimizes these effects, resulting in a more uniform electrochemical response. Furthermore, the improved sodium discrimination may be attributed to a more consistent distribution of ionophores within the membrane, which enhances interference-response screening toward K^+^ over competing monovalent ions.

Regarding urea, which is a neutral molecule and does not directly participate in ion-exchange processes, the observed response is likely related to indirect effects on the solution properties (e.g., ionic strength, viscosity, and membrane hydration) rather than direct ion-exchange processes. The comparable behavior observed for both sensor types suggests that urea does not significantly compromise the intrinsic response mechanism to a single interferent, but rather affects the measurement environment.

The reproducibility analysis (Table 4) revealed a higher variability for AJP sensors compared to commercial devices. This result can be attributed to the current optimization stage of the AJP deposition process, particularly for the polymeric ion-selective membrane, where parameters such as ink viscosity, atomization conditions, and solvent evaporation dynamics play a critical role.

Similar fabrication-related variability has been reported in solid-contact ion-selective electrodes prepared through alternative membrane-deposition strategies, where ionophore distribution, solvent evaporation dynamics, and membrane microstructure significantly influence reproducibility and batch-to-batch uniformity [2].

Unlike industrial screen printing, which benefits from highly standardized and mature processes, AJP fabrication is still undergoing parameter refinement. Nevertheless, the relatively low Nernst deviation and the consistently faster response times indicate that the fundamental electrochemical performance remains robust. These findings suggest that further optimization of the printing parameters could significantly improve batch-to-batch reproducibility without compromising the advantages in response dynamics.

Overall, the results demonstrate that AJP enables a higher degree of control over sensor architecture, which directly translates into improved dynamic performance and more stable interference-response behavior. This capability is particularly relevant for the development of next-generation wearable and miniaturized sensing platforms, where both performance and manufacturability must be carefully balanced.

## 5. Conclusions

This work demonstrates that Aerosol Jet Printing (AJP) is a promising fabrication strategy for ion-selective electrodes, enabling the fabrication of miniaturized and customizable K^+^-selective sensors. The AJP-printed devices exhibited near-Nernstian sensitivity, faster response times, and improved stability in the presence of sodium interference compared to commercial screen-printed electrodes.

This improvement is limited to Na^+^, as the AJP sensors showed comparable behavior toward NH_4_^+^ and a higher sensitivity to urea than the commercial KION electrodes.

However, the higher variability observed in the reproducibility tests and the performance deviations under interference conditions indicate that the AJP sensors are not yet fully comparable to commercial devices in terms of robustness and batch-to-batch uniformity. Despite their functional and consistent potentiometric behavior, AJP-printed electrodes showed higher variability than commercial sensors (RSD = 30.12% vs. 18.45%). These findings highlight both the potential of AJP and the need for further optimization of membrane deposition and printing parameters to improve batch-to-batch reproducibility.

Overall, the findings support the use of AJP as a versatile platform for next-generation ion-selective sensors, particularly in wearable, portable, and environmental monitoring applications where rapid response, miniaturization, and manufacturing flexibility are essential.

## Figures and Tables

**Table 1:** Types of sensors used in the study.

Sensor Type	Conductive Substrate	Conductive Materials	PVC Membrane	Deposition Method
KION	Carbon	Conductive additives (e.g., PEDOT/MWCNTs, not declared)	Provided by the manufacturer	Screen printing
AJP	Carbon	MWCNTs (printed)	Printed	Aerosol Jet Printing

**Table 2:** Quantitative data for sensor analysis & interference tests.

Additions	Time (s)	Added Volume (mL)	Concentration of Added Solution (M)	Concentration of Final Solution (M)
1	0	0.01	10^−2^	10^−6^
2	100	0.09	10^−2^	10^−5^
3	200	0.026	0.34	10^−4^
4	300	0.266	0.34	10^−3^
5	400	0.92	1	10^−2^

**Table 3:** Sensor parameters after four tests (repeatability tests).

Sensor	Slope (mV/dec)	RSD (%)	Nernst Deviation (%)	R^2^	t90 (s)
KION	57.28 ± 5.07	8.86	3.18	0.9742	26.44
AJP	57.91 ± 5.07	8.75	2.11	0.9554	23.53

**Table 4:** Reproducibility parameters for the thirty sensors. Fifteen for each sensor type.

Sensor	Slope (mV/dec)	RSD (%)	ND (%)	R^2^	t90 (s)
KION	61.75 ± 11.39	18.45	4.38	0.9755	31.14
AJP	56.41 ± 16.99	30.12	4.64	0.9598	21.10

**Table 5:** Parameters for the two types of sensors in the interference tests.

Type of Sensor	Analyte	Sensitivity (mV/dec)	R^2^
KION	K^+^	57.28 ± 5.07	0.9742
	**NH_4_^+^**	20.60 ± 4.86	0.884
	**Na^+^**	33.95 ± 8.95	0.936
	**Urea**	5.89 ± 2.77	0.847
AJP	K^+^	57.91 ± 5.07	0.9554
	**NH_4_^+^**	19.64 ± 7.35	0.942
	**Na^+^**	24.37 ± 1.39	0.936
	**Urea**	10.46 ± 3.71	0.817

## Data Availability

The data supporting the findings of this study are available from the corresponding author upon reasonable request.
